# [18F]FDG PET/CT Radiomics in Untreated Breast Carcinoma: A Review of the Current State and Future Directions

**DOI:** 10.3390/diagnostics15172231

**Published:** 2025-09-03

**Authors:** Alexandru Mitoi, Raluca-Mihaela Mititelu, Cosmin Medar, Ciprian Constantin, Vlad-Octavian Bolocan, Ioan-Nicolae Mateș

**Affiliations:** 1 Doctoral Program Studies, University of Medicine and Pharmacy “Carol Davila”, 050474 Bucharest, Romania; alexandru.mitoi@drd.umfcd.ro (A.M.); vlad-octavian.bolocan@drd.umfcd.ro (V.-O.B.); 2 Department of Nuclear Medicine, Carol Davila University of Medicine and Pharmacy, 010825 Bucharest, Romania; raluca.mititelu@umfcd.ro; 3 Clinic of Nuclear Medicine, University Emergency Central Military Hospital “Dr. Carol Davila”, 010825 Bucharest, Romania; 4 Department of Fundamental Sciences, Faculty of Midwifery and Nursing, University of Medicine and Pharmacy “Carol Davila”, 050474 Bucharest, Romania; 5 Clinical Laboratory of Radiology and Medical Imaging, Clinical Hospital “Prof. Dr. Theodor Burghele”, 050664 Bucharest, Romania; 6 Department of Diabetes and Nutrition, Medicine Faculty, Titu Maiorescu University, 040051 Bucharest, Romania; 7 General and Esophageal Surgery Clinic, Sfanta Maria Clinical Hospital, Carol Davila University of Medicine and Pharmacy, 020021 Bucharest, Romania; ioan.mates@umfcd.ro

**Keywords:** PET/CT, [18F]FDG, breast, carcinoma, radiomics, ER, PR, HER2

## Abstract

**Background/Objectives**: [18F]FDG PET/CT radiomics could improve risk stratification in untreated breast carcinoma. **Methods**: PubMed Central was accessed for full-text English articles (2015–2025) evaluating radiomic features from pretreatment [18F]FDG PET/CT. The Newcastle-Ottawa Scale (NOS) was used to evaluate the risk of bias. **Results**: Seven studies (1394 patients with a median cohort of about 150 patients) met the inclusion criteria. Radiomics outperformed conventional metabolic measures at predicting pCR to NAC (with the best AUC 0.83 when combining intra- and peritumoral features); differentiating molecular subtypes (AUC 0.856 luminal vs. non-luminal; 0.818 HER2+ vs. HER2−, and 0.888 triple negative vs. others); and assessing androgen receptor (AR) expression. No additional value was found for ER/PR status. Age influenced SUV and texture metrics, especially in triple-negative lesions. Methodological variation was notable: all studies were retrospective, the majority were single-center, only two provided external validation with different protocols of acquisition and segmentation, and at least four distinct software platforms were used for feature extraction and statistical analysis. **Conclusions**: [18F]FDG PET/CT radiomics shows good potential for predicting neoadjuvant response and molecular profile in breast cancer. However, small, diverse cohorts and non-standardized methodologies limit the evidence. Prospective multicenter studies with standardized acquisition, segmentation and feature extraction are required before clinical use.

## 1. Introduction

According to the World Health Organization, in 2024, nearly 2.3 million new cases of breast cancer were diagnosed globally, making it the second most common cancer after lung cancer [1]. The incidence rate is higher in developed countries, and the prevalence of breast cancer varies considerably by race and ethnicity [2,3]. Additionally, with approximately 670,000 deaths worldwide, it is the 4th leading cause of cancer mortality [1]. The incidence of breast carcinoma peaks in the 7th decade, affecting approximately 1 in 25 women. However, approximately 14% of breast cancer cases occur in women aged 40, which represents 1 in 65 women [4].

Lifestyle, environmental factors, and socio-psychological factors are associated with its occurrence. Recent studies have shown that less than 10% of breast cancer cases are attributed to hereditary genetic mutation, and approximately 30% are the result of risk factors [2,3,5]. Among the most common risk factors are female sex, nulliparity, advanced age, family history of breast cancer in first-degree relatives, hormone replacement therapies, exposure to ionizing radiation, obesity, and alcohol consumption [2,5,6,7]. Certain factors provide protection against the onset of breast cancer, with concrete data regarding the benefits of parity and multiparity at ages under 35, an extended breastfeeding period, maintaining a normal body mass index (BMI), and regularly engaging in physical activities [5].

From a histopathological perspective, the two most common subtypes of breast neoplasms are invasive carcinoma of no special type (NOS), previously known as ductal carcinoma, accounting for approximately 75% of cases [8], and invasive lobular carcinoma, up to 15% [9]. The other, much rarer subtypes of breast cancer (cribriform, medullary, mucinous, tubular, etc.) account for approximately 5% of cases, each having a specific prognosis [8].

To establish a prognostic score and develop an appropriate therapeutic plan, it is necessary to classify tumors into subtypes based on histological and immunohistochemical characteristics.

The eighth edition of the American Joint Committee on Cancer (AJCC) includes two staging systems [10]:

1. The anatomic stage, which includes the characteristics of the primary tumor (T), lymph node invasion (N), and distant metastases (M),
2. The prognostic stage, which incorporates the degree of tumor differentiation, hormonal receptor status, estrogen receptors (ER), progesterone receptors (PR), human epidermal growth factor receptor 2 (HER2) expression, and multigene test results alongside the anatomic stage.

A frequently used classification of breast carcinoma is based on the status of ER, PR, and HER2, and divides it into five subtypes: luminal A, luminal B HER2-positive, luminal B HER2-negative, HER2-positive (non-luminal), and triple-negative, each with its own diagnostic method, treatment, and different prognosis [11].

Other factors that influence prognosis include the Ki-67 cell proliferation marker [12], the number of affected regional lymph nodes, the degree of differentiation, and the presence of peritumoral vascular invasion [13].

### 1.1. Positron Emission Tomography Combined with Computed Tomography Using 18F-Fluoro-2-deoxy-D-glucose ([18F]FDG PET/CT)

[18F]FDG PET/CT is increasingly used in patients with breast cancer for initial staging regardless of histological subtype, evaluation of response to systemic treatment, assessment of recurrences (locoregional or distant), and post-therapeutic restaging, and it can also guide the radiotherapy and surgical plan [14,15].

In the diagnosis of primary breast carcinoma, most studies report a sensitivity of over 50% and a specificity of over 70%, these being closely related to the histopathological subtype and the size of the tumor (clinically detectable tumors reaching a sensitivity of over 90%) [15].

Since some benign tumors (including fibroadenomas), physiological breast tissue (especially glandular tissue in breastfeeding patients), or certain inflammatory processes may exhibit avidity for [18F]FDG, PET/CT examination is not recommended for the primary detection of breast carcinoma [16,17].

The American National Comprehensive Cancer Network (NCCN) guidelines recommend [18F]FDG PET/CT investigation starting at stage I (tumor larger than 1 cm and HER2 positive or triple negative) and stage IIA, regardless of histopathological subtype and prognosis [18].

[18F]FDG PET/CT is superior to bone scintigraphy and contrast-enhanced CT and is comparable to MRI in terms of specificity and sensitivity in detecting bone metastases [17,19,20].

The major advantage of [18F]FDG PET/CT is that, unlike classical imaging techniques, it has a higher accuracy in detecting distant metastases, with a sensitivity of over 97% and a specificity of over 91% [21]. Thus, it has a major impact on therapeutic management.

A significant number of patients with stage II-III breast carcinoma receive neoadjuvant chemotherapy. The maximum of the standardized uptake value (SUVmax) is an important predictor of treatment response, especially when used in monitoring lymph nodes [22]. Standardized uptake value (SUV) and SUVmax variations can differentiate responders to systemic treatment and identify pathological complete responses (pCR) [22,23]. Recent studies have shown that the SUVmax value after treatment is closely connected to complete response (pCR), especially in patients with HER2+ and triple-negative breast cancer, where a decrease in SUVmax values suggests a better outcome for those receiving additional chemotherapy [22].

### 1.2. Radiomics

Radiomics is a new scientific field used in medical imaging that employs advanced algorithms for analyzing imaging data. Radiomics succeeds in extracting characteristic and quantifiable data from imaging examinations [24].

It identifies certain subtle quantitative features from an image or a designated region/volume of interest (ROI/VOI). Pattern recognition algorithms are used to generate a numerical set that quantitatively describes a specific geometric or physical property of the region of interest [25]. The quantitative and textural values of tumor uptake obtained through [18F]FDG PET/CT, such as the standardized uptake value (SUV), metabolic tumor volume (MTV), and total lesion glycolysis (TLG), serve as important prognostic indicators, being associated with disease-free survival or overall survival [26,27,28,29].

Triple-negative breast cancer frequently exhibits increased avidity for 18F-FDG [27], with studies establishing associations between SUVs and prognosis, tumor differentiation grade, or Ki-67 [29].

The radiomic characteristics of [18F]FDG PET/CT significantly correlate with certain clinical and biological parameters of breast carcinoma, including the degree of differentiation, ER/PR receptor status, and HER2 expression. Recent studies have investigated the potential to predict the expression of the Ki-67 proliferation marker using a radiomic technique, successfully differentiating cases with low Ki-67 from those with high values [25,29].

## 2. Materials and Methods

We conducted a review of the specialized literature regarding the application of radiomics techniques in [18F]FDG PET/CT imaging of untreated breast carcinoma.

The main objective of this review was to evaluate the performance of radiomic features as advanced imaging biomarkers, complementary characteristics to standard metabolic markers [SUVmax, average SUV within a fixed-size volume of 1 cm^3^ sphere (SUVpeak), standardized uptake value normalized to lean body mass—maximum (SULmax)] in [18F]FDG PET/CT in untreated breast cancer. The study aims to assess the contribution of these advanced imaging features to the development of new diagnostic and predictive models.

The secondary objectives included:

- highlighting correlations between radiomic data and the tumor histopathological profile (ER, PR, HER2, Ki67);
- exploring the potential of radiomics for personalizing diagnostic and treatment strategies in breast carcinoma.

The study was based on scientific articles published between 1 January 2015 and 1 January 2025, accessible in the PubMed Central^®^ database (U.S. National Library of Medicine, Bethesda, MD, USA), in English, with full text available for free. We used combinations of terms such as “radiomics,” “PET CT,” “breast,” “carcinoma,” and “18F FDG.” Title and abstract screening was the first step in the study selection process.

We designed this work as a narrative, focused review, in which we intentionally incorporated certain structured elements typically used in systematic reviews such as a PRISMA-style flow diagram and Newcastle–Ottawa Scale (NOS, Ottawa Hospital Research Institute, Ottawa, ON, Canada) appraisal, to enhance transparency and traceability, while not aiming to fulfill the requirements of a full systematic review.

The evaluation of the articles was carried out based on a well-established plan, and the decisions for inclusion and exclusion were documented to ensure the reproducibility of the process.

Only original studies that explicitly investigated radiomic features extracted from [18F]FDG PET/CT images of primary breast tumors in untreated breast cancer patients (without surgical intervention, chemotherapy, radiotherapy, hormone therapy, or immunotherapy) were included.

The exclusion criteria were represented by a lack of direct information and results directly related to PET/CT radiomics in untreated breast carcinoma, use of other radiotracers besides [18F]FDG, the absence of full-text articles, review articles, studies that focused primarily on axillary lymph node status (including sentinel node evaluation) or on other anatomical sites or those without original data. This approach ensured the selection of the most relevant scientific data for our analysis.

For the rigorous evaluation of methodological quality, we used the Newcastle-Ottawa Scale, a validated tool for analyzing the risk of bias in observational studies. Studies with an NOS score ≤ 5 were considered at high risk of bias and were excluded. Articles with an NOS score > 5 were considered eligible, classifying their quality as follows: 6–7 moderate risk, 8–9 low risk of bias [30].

This review synthesizes primary studies of baseline [18F]FDG PET/CT radiomics in untreated breast carcinoma and, in brief, considers the typical workflow, local acquisition under site-specific protocols, VOI segmentation, feature extraction, and predictive modeling toward clinicopathologic endpoints.

## 3. Results

After applying the search technique described in the Materials and Methods section, the PubMed query “18F-FDG PET/CT AND radiomics AND breast carcinoma” identified 17 potentially relevant articles.

**Figure 1:**
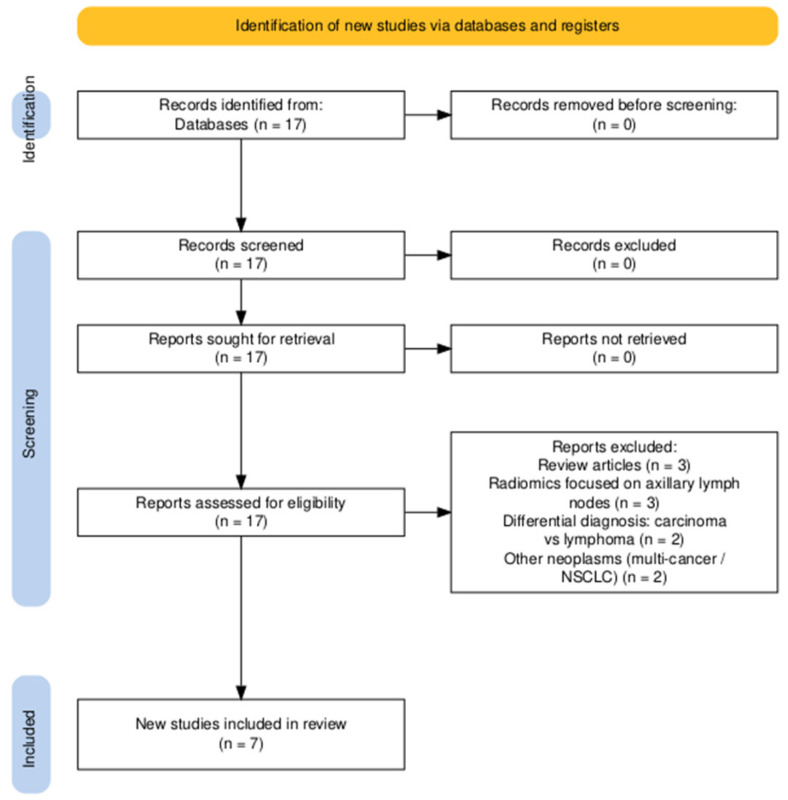
PRISMA-style diagram.

After analyzing them, 10 articles were excluded from our study as follows. Three articles were of the review type, three other articles focused on radiomic information related to axillary lymph nodes, two articles aimed at differentiating breast carcinoma from other pathologies (breast lymphoma), and two examined the radiomic characteristics of different types of neoplasms. As a result, only 7 articles are considered for the final analysis, as shown in the PRISMA-style diagram (Figure 1) [31].

To help track the quality of the studies we included, we have shown the parts of the NOS score in Table 1 and Table A1 from Appendix A.

Even though we did not include articles with a high risk of bias, the fact that 71% (5 articles) presented a moderate risk means that the results of the analysis should be interpreted with caution. However, there are common strengths, such as diagnosis confirmed by histopathological result, patients presented for [18F]FDG PET/CT with pre-treatment breast carcinoma, and most used open-source image analysis software (PyRadiomics, LIFEx, 3D Slicer, and CGITA, shown in Appendix A, Table A2) with semi-automated image processing.

Three of these studies investigated the potential of radiomics in predicting complete pathological response after neoadjuvant chemotherapy [32,37,38]. The results varied considerably: individual characteristics, such as the normalized distance between the center of the SUVpeak volume and the geometric center of the tumor (NHOCpeak), showed moderate predictive value (AUC up to 0.71) [38], while complex models that integrated both intratumoral and peritumoral characteristics achieved superior accuracy, with AUCs up to 0.83 [37]. These data highlight the importance of radiomic heterogeneity descriptors and the benefit of combining multiple features, compared to using conventional PET parameters alone.

Two studies focused on the classification of molecular subtypes [34,35]. Radiomic texture and shape parameters allowed for robust differentiation between luminal and non-luminal tumors (AUC 0.81), and between triple-negative and non-triple-negative cancers (AUC 0.88) [34]. Regarding receptor profiles, radiomic signatures such as sphericity and Gray-level co-occurrence matrix (GLCM) contrast derived from CT have proven useful for the non-invasive prediction of androgen receptor expression (AUC 0.83) [36]. However, for estrogen and progesterone receptors, no significant additional benefit was found compared to standard [18F]FDG PET/CT parameters [35].

Another study highlighted the influence of patient-related factors, demonstrating that both SUVs and textural characteristics are affected by age, particularly in the case of triple-negative breast cancers [33]. This finding suggests the need to include age as a control variable in radiomic models.

Overall, while methodological heterogeneity prevented a quantitative meta-analysis, the available data consistently support the fact that PET/CT radiomics with [18F]FDG can provide complementary biological and clinical information about tumor heterogeneity, therapeutic response, and molecular profile in untreated breast carcinoma.

The key information from the seven included articles was cataloged based on objectives, sample size, statistical and imaging analysis methods used, results, and conclusions obtained, which are summarized in Table 2. We chose to extract the relevant data in a tabular format to facilitate direct comparison between studies. For more detailed information on the selected articles, please refer to Table A3 from Appendix A.

## 4. Discussion

This review provides an updated perspective on the use of [18F]FDG PET/CT radiomics in characterizing untreated breast carcinoma. The main contribution was the exclusive inclusion of studies that analyzed only pretreatment cohorts, avoiding confounding effects of prior therapy.

### 4.1. Methodological Limitations of the Analyzed Studies

Most of the studies had the same methodological limitations. All 7 studies were retrospective, 5 of them were monocentric, only 2 reported an external validation set, and none applied the Image Biomarker Standardisation Initiative (IBSI) standard for all stages (acquisition, reconstruction, segmentation, and post-processing). The cohorts were relatively small, with a median size of approximately 150 patients; additionally, 57% (4 studies) had fewer than 155 patients. There were also variations between the [18F]FDG PET/CT acquisition protocols. Additionally, we encountered diversity in the software used for image processing, with four software programs being utilized (CGITA, LIFEx, PyRadiomics, and 3D Slicer).

In the analyzed radiomic studies, the VOI (volume of interest) was defined either by an absolute threshold SUV = 2.5 [32], either relative to the tumor activity with a threshold of 40% of SUVmax [33,35] or 42% of SUVmax [34] or through manual segmentation by experienced doctors, followed by the application of a threshold of 40% of SUVmax, which included both MTV and TLG [36]. In the study by Hou et al. [37] the VOI was extended with a 2 mm peritumoral margin, and in Hong et al. [38] they only calculated the spatial radiomic indices NHOC and NHOP (the distance from the SUVpeak to the tumor center and the tumor mass periphery).

Practically, in the absence of standardized segmentation protocols, incomparable VOIs and radiomic features are generated, limiting the quantitative synthesis of the results.

Despite these limitations, studies suggest that radiomics has real potential in personalizing treatment strategies in breast cancer. Possible applications include the prediction of pCR in NAC [32,37,38], estimating the risk of recurrence and progression-free survival [32,38], classification of molecular subtypes [34,37], non-invasive evaluation of the androgen receptor [36], and of the ER/PR hormone receptors [35]. Age, especially in the case of triple-negative carcinoma, can influence the performance of radiomic markers [33].

### 4.2. Prediction of pCR in NAC

According to the specialized literature, pCR after NAC is a significant prognostic indicator in patients with breast carcinoma [39].

Based on a cohort of 73 patients with locally advanced breast carcinoma, Ha et al. [32] demonstrate that the metabolic texture of the tumor can predict pCR. Starting from the segmentation of the lesion based on an SUV of 2.5, they extracted 109 radiomic features, and based on these, 3 tumor clusters were formed. In their study, tumor cluster II (increased SUVmax values and heterogeneity) was the one that had the majority of cases with pCR. Tumor cluster I, which had a low SUVmax and heterogeneity, also displayed a high rate of tumor recurrences. High metabolic heterogeneity can be an early sign of a favorable response to neoadjuvant chemotherapy treatment.

Hou et al. [37] demonstrated the value of adding peritumoral tissue to the model. In a series of 190 pre-NAC patients, over 3800 radiomic features were extracted from the VOI and from a special segmentation that also included a 2 mm peritumoral area. After wavelet filtering and using the least absolute shrinkage and selection operator (LASSO), an SVM that combined five intratumoral and five peritumoral features had an AUC = 0.83, with sensitivity and specificity over 79%, surpassing models based exclusively on tumor tissue. For molecular subtypes, performance was maintained with AUC values ranging between 0.86 and 0.92, suggesting that peritumoral tissue complements intratumoral radiomic data.

Complementary to the textural radiomic indices, Hong et al. [38] introduced a spatial index of the metabolic “hot spot.” In a multicentric cohort of 135 cases, the NHOCpeak index (the normalized distance between the SUVpeak volume center and the tumor geometric center) predicted pCR with an AUC of 0.69–0.71 and was able to stratify prognosis: patients with NHOCpeak ≤ 0.27 achieved a 92% progression-free survival at five years, compared to 67% for higher values. The parameter remained independent of SUVmax, MTV, and TLG, indicating that not only the intensity but also the position of the hypermetabolic focus influences the response to NAC.

The three studies suggest a stepwise model for predicting pCR: increased heterogeneity signals treatment sensitivity, and the addition of peritumoral features increases accuracy by approximately 10% AUC, reflecting the role of the tumor stroma microenvironment and local inflammation. Finally, the spatial indices of the “hot spot” refine the models and provide them with long-term prognostic value.

### 4.3. Characterization of the Molecular Profile and Hormonal Receptor Status

Within a multicentric study on a cohort of 273 patients, Liu et al. [34] demonstrate that textural and metabolic indices can predict the molecular type of breast tumors. With 1710 extracted radiomic features, the LASSO function reduced the number of variables relevant for the analysis to nine. The obtained radiomic score identified tumor subtypes (luminal vs. non-luminal; HER+/− and triple-negative vs. non-triple-negative) with AUC 0.856 for luminal versus non-luminal, 0.818 for HER2+, and 0.888 for triple-negative versus non-triple-negative. Internal analysis of Hou et al. [37] confirms the direction: in their test set, models based on 10 features (intra- and peritumoral) maintained AUC 0.86–0.92 for the same pairs of tumor subtypes.

In the series of 153 patients by Araz et al. [35], the selection process (binary logistic regression analysis) reduced the initial set to seven robust markers: SUVmean/max/peak, MTV, TLG, GLZLM-LZE, and GLRLM-GLNU, but none proved to be independent of ER/PR, thus unable to predict hormonal status. On the other hand, the androgen receptor was predicted radiomically. Jia et al. [36] combined three metabolic and textural markers (MTV, SphericityCT, and GLCM-ContrastCT) into a logistic model, obtaining AUC values of 0.832 and an odds ratio of 9 for GLCM-ContrastCT. Sensitivity and specificity exceeded 75%, with good calibration (Hosmer-Lemeshow *p* > 0.05). The increased MTV value was inversely correlated with AR expression, suggesting that a high metabolic volume likely indicates AR-negative tumors.

Radiomics manages to discriminate molecular subtypes with an accuracy greater than 0.80, consistent performance across a model rich in features (Liu et al. [34]) and a restrictive model (Hou et al. [37]). In the case of ER/PR hormone receptors, radiomic models do not provide additional data beyond SUV and TLG, although hormone-dependent tumors exhibit lower SUVs [35]. In contrast, AR status can be reasonably quantified through a mixed combination of metabolic and textural markers, which opens the perspective for non-invasive selection of patients eligible for anti-androgen therapies.

### 4.4. Individual Patient Parameters, Biological and Methodological Variability

Boughdad et al. [33] had the only study in our analysis that systematically quantified the impact of patient characteristics. The authors analyzed a cohort of 522 patients, who were divided into three groups: PRE (under 45 years old), PERI (45–54 years old), and POST (over 55 and under 85 years old), and found a progressive decrease in SUVmax/mean/peak (r_Spearman approximately −0.4; *p* < 0.001) alongside increased homogeneity, SRE, and HGZE. The effect was particularly pronounced in the triple-negative subtype (interaction “age × TN” *p* = 0.02). At ages over 55, high LRE (long run emphasis) values indicated a more uniform tumor texture, potentially related to stromal involution. The other six studies did not include age, menopause, or BMI in the predictive models. This partially explains the performance divergences between the higher SUVs observed in the younger cohort of Ha et al. [32] compared to the moderate values from the older series of Hong et al. [38].

The technical differences arise from the different protocols for acquiring [18F]FDG PET/CT images, the segmentation methods, and the use of various extraction software. Ha et al. [32] used CGITA, Boughdad et al. [33], Araz et al. [35], Jia et al. [36] and Hong et al. [38] analyzed the images in LIFEx, and Hou et al. [40] resorted to the tandem of 3D Slicer and PyRadiomics, the latter being the only combination explicitly compliant with the IBSI standard (if the parameters are set correctly). CGITA and the LIFEx versions used in the initial studies do not automatically report IBSI post-processing.

In the included studies, artificial intelligence was predominantly utilized for feature selection (e.g., LASSO) and classification (e.g., SVM or logistic regression) employing 10-fold cross-validation. Performance metrics were summarized using AUC, accuracy, and sensitivity/specificity, supplemented by occasional calibration, decision-curve analysis, and inter-observer reproducibility (ICC). Since all of the groups were local and used site-specific [18F]FDG PET/CT protocols, future developments should use SUVmax/MTV/TLG as a standard, report AUCs with confidence intervals and basic calibration, show that the results can be repeated, and, if possible, look for outside validation.

Thus, radiomic characteristics transform molecular imaging into a faithful indicator of tumor biology, with the potential to reduce the number of invasive interventions and personalize therapy for patients with breast carcinoma.

### 4.5. Strengths and Limitations of the Study

This review has several limitations. Although the structure of the analysis largely adheres to the principles of a systematic review, a PRISMA protocol was not registered, which limits methodological transparency. The small number of included articles (*n* = 7) found in a single database (PubMed Central^®^; full-text free as a filter) reflects a rigorous selection of cohorts with untreated patients but limits the applicability of the conclusions and the possibility of conducting a statistical meta-analysis. The heterogeneity of methodological approaches, the lack of external validations, and the absence of a standardized quantitative comparison (e.g., between AUCs) constitute additional limitations in consolidating universal conclusions.

Since we aimed to synthesize primary research, we did not extend the work to a market overview of commercial radiomics products or a regulatory evaluation. The studies included did not utilize FDA-cleared or CE-marked radiomics software for breast [18F]FDG PET/CT; instead, they employed research-oriented tools.

After an updated query of the PubMed database (terms: radiomics, [18F]FDG PET/CT, breast carcinoma), we identified several reviews, including the studies by Urso et al. [29] and Hwang et al. [41], but no study is limited only to data on radiomic markers in untreated breast carcinoma. Thus, to the best of our knowledge, the present article fills this gap in the literature by focusing strictly on the value of radiomics in pretreatment breast carcinoma, before treatment alters the molecular features.

Overall, three converging directions can be outlined from the seven studies analyzed. Firstly, radiomic heterogeneity descriptors, especially when combined with peritumoral features or spatial indices, offer added predictive and prognostic value compared to conventional PET parameters, particularly for evaluating pCR after NAC.

Secondly, the discrimination of molecular subtypes is robust regardless of the methodology, while the prediction of ER/PR status remains limited; in contrast, androgen receptor status represents a promising radiomic target.

Thirdly, both patient-related variables (especially age, relevant in the case of triple-negative tumors) and methodological variability (acquisition protocols, segmentation strategies, analysis software) significantly influence the results, highlighting the urgent need for standardization. Taken together, these observations outline the potential of [18F]FDG PET/CT radiomics to complement conventional imaging and also define the methodological steps necessary for true clinical translation.

## 5. Conclusions

The application of radiomics to [18F]FDG PET/CT images in untreated breast cancer provides a way to characterize breast tumors using non-invasive techniques, which complement conventional histopathological evaluation. Mainly, the analyzed studies suggest that radiomic features can be used to predict the pCR response to NAC, surpassing conventional metabolic markers, the evaluation of androgen receptor status, and tumor subtype classification. Radiomics loses its usefulness for ER/PR hormone receptors and remains sensitive to demographic variables, particularly age.

Most of the radiomic models analyzed in our study demonstrated good performance (AUC > 0.80), but the generalization of these results is limited by the lack of external validation and variations in segmentation and feature extraction methodology, as well as the small sizes of the cohorts.

For the integration of [18F]FDG PET/CT radiomics into clinical practice, we recommend a structured approach based on a clear protocol: complete standardization of acquisition and segmentation according to the IBSI guideline, the possibility of multicenter validation, and the implementation of automated analysis platforms based on artificial intelligence, integrated into the PACS system.

In parallel, future studies must rigorously evaluate the value that radiomic features offer beyond conventional PET (metabolic) parameters in well-defined clinical scenarios, reporting performance according to a standardized format. Only by following these steps (IBSI standard, external validation, and standardized reporting) can [18F]FDG PET/CT radiomics be rationally adopted by the medical community in the management of breast cancer.

## Figures and Tables

**Table 1:** Evaluation of the quality of the included articles using the Newcastle-Ottawa Scale.

Author	Selection (0–4)	Comparability (0–2)	Result (0–3)	Total NOS (0–9)
Ha et al. [32]	3	1	2	6
Boughdad et al. [33]	3	1	2	6
Liu et al. [34]	4	2	2	8
Araz et al. [35]	3	1	2	6
Jia et al. [36]	3	1	2	6
Hou et al. [37]	4	1	2	7
Hong et al. [38]	4	1	3	8

**Table 2:** Summary of identified studies on PET/CT radiomics in untreated breast carcinoma.

	Ha et al. (2017) [32]	Boughdad et al. (2018) [33]	Liu et al. (2021) [34]	Araz et al. (2022) [35]	Jia et al. (2023) [36]	Hou et al. (2024) [37]	Hong et al. (2024) [38]
Objective	[18F]FDG PET/CT radiomics for the characterization of LABC and the prediction of pCR	Age influence on metabolic and radiomic parameters in BC and healthy breast tissue.	The use of radiomics for the classification of molecular subtypes of breast cancer	Prediction of hormonal receptor status through radiomics	Predicting AR expression using radiomic characteristics	Evaluation of intratumoral and peritumoral radiomic characteristics for assessing pCR response after NAC	NHOC and NHOP predict breast carcinoma NAC progression-free survival and rPC
Study type	Retrospective Monocentric	Retrospective Monocentric	Retrospective Multicentric	Retrospective Monocentric	Retrospective Monocentric	Retrospective Monocentric	Retrospective Multicentric
No. of patients	73	522	273	153	48	190	135
Image and radiomic analysis	CGITA version 1.4 109 TI	LIFEx software 4 indices based on histograms 31 TI	ImageJ 1.50i and MATLAB 1710 TI (855 specific to PET, respectively 855 based on CT)	LIFEx 42 TI: first, second, and higher-order parameters	LIFEx v7.0.0 80 TI	3D Slicer software 4.11.20200930 and PyRadiomics (IBSI) 1932 intratumoral TI 1932 peritumoral TI	LIFEx v. 7.6.0 compliant (IBSI) 4 classic metabolic markers 4 radiomic markers derived from distance
Statistical analysis	MedCalc 14.8.1 and R version 3.2.3 Unsupervised clustering, Pearson, Kruskal–Wallis, χ^2^, uni/multi logistic regression, Kaplan–Meier, Cox, Bonferroni	IBM SPSS Statistics v22.0 Test ANOVA Test Bonferroni Hochberg Spearman	R 3.2.2 Wilcoxon rank-sum, coefficient inter feature R, LASSO-Cox, Rad score, ROC, 10 × 10 fold cross validated	WEKA 3.7 and SPSS 11.5 Mann–Whitney U test, binary logistic regression, ROC, Hoeffding tree, J48, multilayer perceptron, 10-fold cross- validation	IBM SPSS 26.0, Python version 3.11, MedCalc and R version 4.2.1 Kolmogorov–Smirnov tests, Levene, *t*-test, Mann–Whitney U, χ^2^, Fisher, Pearson, multivariate logistic, ROC, 10-fold cross-validated	R software 4.3.1 and Python 3.7.9 Pearson and Spearman correlation, *t*-test, LASSO, ROC-AUC and DeLong test, χ^2^, SMOTE. SVM, KNN, LR, NB classifiers	MedCalc Statistical v. 22.021 Mann–Whitney U, Kruskal–Wallis, Spearman, ROC-AUC, logistic regression, cox, Kaplan–Meier, log-rank test
Radiomic features	SUVmax, MTV, TLG, NL_EntropyGLCM, NL_HomogeneityGLCM, ZPGLSZM, Skewness	SUVmax SUVmean SUVpeak 12 TI including: Homogeneity SRE HGZE	LASSO reduced the TI set from 1710 to 9 (3 for each breast cancer classification). The individual TI were not listed in the main text of the study.	7 statistically relevant TI: SUVmean, SUVmax, SUVpeak, GLZLM LZE, TLG, MTV, and GLRLM-GLNU.	MTV, SHAPE-sphericityCT, and GLCM-contrastCT; GLCM-contrastCT being the strongest predictor (OR-9, *p* = 0.018)	Include 10 wavelet-filtered features, Laplacian-Gaussian (5 intratumoral and 5 peritumoral) selected by LASSO. The features demonstrated robustness in segmentation (ICC > 0.75) and were validated with SMOTE.	4 radiomic features derived from distance: NHOCmax/peak and NHOPmax/peak
Results	Cluster TC II (increased SUVmax values and heterogeneity) achieved pCR; Cluster TC I = high risk of recidivism. Significant differences	SUVmax/mean /peak decrease with age Homogeneity and LRE increase with age. Significant differences, especially in the TN subtype (*p* < 0.050)	Mean AUC 0.856 Luminal vs. Non-Luminal 0.818 HER+ vs. HER2− 0.888 TN vs. Non-TN	No radiomic feature predicted hormone receptor status	The final logistic model based on MTV, SHAPE-sphericityCT, and GLCM-contrastCT can predict androgen receptor status with an AUC of 0.83 and sensitivity and specificity > 75%.	Intratumoral and peritumoral SVM had the best prediction of pCR, with an AUC of 0.83, significantly outperforming KNN, LR, and NB. Molecular subtype models maintained high performance (AUC 0.86–0.92).	NHOCmax and NHOCpeak AUCs of 0.71 and 0.69 predict NAC pCR better than SUVmax/peak, MTV, and TLG. Low NHOCmax and NHOCpeak enhance pCR risk. Low NHOCpeak (≤0.27)—92% progression-free survival at 5 years vs. 67%.
Conclusions	Pre-treatment radiomics correlates with Ki-67, predicts pCR and recurrence risk.	Age influences SUV and TI, especially in triple-negative BC. Radiomic studies should include age.	Radiomic models are superior to classic [18F]FDG PET/CT parameters in identifying the molecular subtype.	Radiomics did not predict the hormonal receptor status. Patients with hormone receptors had lower SUVs	The model built on clinico-pathological data and [18F]FDG PET/CT radiomic features can predict AR presence in BC.	The combination of intra- and peritumoral features increases performance in predicting pCR.	NHOCpeak can predict pCR and the response to NAC. Increased NHOCpeak is associated with a worse prognosis.

Legend: BC—breast carcinoma; LABC—locally advanced breast carcinoma; pCR—pathological complete response; CGITA—Chang-Gung Memorial Hospital, Taiwan; TC—tumor cluster; TI—textural index; SVM—Support Vector Machine; KNN—k-nearest neighbors; LR—logistic regression; AUC—area under the ROC curve; ROC—receiver operating characteristic curve; LASSO—least absolute shrinkage and selection operator; SMOTE—synthetic oversampling technique for the minority class; NHOCmax—the normalized distance between the voxel with SUVmax and the geometric center of the tumor; NHOCpeak—the normalized distance between the center of the SUVpeak volume and the geometric center of the tumor; NHOPmax—the normalized distance between the voxel with SUVmax and the tumor perimeter; NHOPpeak—the normalized distance between the SUVpeak volume center and the tumor perimeter.

## Data Availability

The original contributions presented in the study are included in the article, further inquiries can be directed to the corresponding authors.

## Abbreviations

The following abbreviations are used in this manuscript:

[18F]FDG	18F-fluoro-2-deoxy-D-glucose
AJCC	American Joint Committee on Cancer
AR	Androgen receptor
AUC	Area under the ROC curve
BC	Breast carcinoma
CGITA	Chang-Gung Memorial Hospital Taiwan
ER	Estrogen receptor
GLCM	Gray-Level Co-occurrence Matrix
HER2	Human epidermal growth factor receptor 2
IBSI	Image Biomarker Standardisation Initiative
KNN	K-nearest neighbors
LABC	Locally advanced breast carcinoma
LASSO	Least absolute shrinkage and selection operator
LR	Logistic regression
MRI	Magnetic resonance imaging
MTV	Metabolic tumor volume
NCCN	American National Comprehensive Cancer Network
NHOCmax	The normalized distance between the voxel with SUVmax and the geometric center of the tumor
NHOCpeak	The normalized distance between the center of the SUVpeak volume and the geometric center of the tumor
NHOPmax	The normalized distance between the voxel with SUVmax and the tumor perimeter
NHOPpeak	The normalized distance between the SUVpeak volume center and the tumor perimeter
pCR	pathological complete response
PET/CT	Positron emission tomography combined with computed tomography
PR	Progesterone receptor
ROC	receiver operating characteristic curve
ROI	Region of interest
SHAPE	Shape-based features
SMOTE	Synthetic oversampling technique for the minority class
SULmax	Standardized uptake value normalized to lean body mass—maximum
SUV	Standardized uptake value
SUVmax	Maximum value of the standardized uptake value
SUVmean	Mean value of the standardized uptake value
SUVpeak	Average SUV within a fixed-size volume of 1 cm^3^ sphere
SVM	Support Vector Machine
TC	Tumor cluster
TI	Textural index
TLG	Total lesion glycolysis
VOI	Volume of interest

## Appendix A

**Table A1:** NOS characteristics.

Study	Selection (Max. 4)	Comparability (Max. 2)	Outcome (Max. 3)	Total (Max. 9)
Ha et al., 2017 [32]	3 points single-centre LABC cohort, consecutive patients no internal control outcome not present at start (pCR not yet occurred)	1 point adjusted for T-stage and Ki-67 but not age or BMI	2 points pCR from pathology follow-up median > 24 months <10% lost (not reported)	6 points
Boughdad et al., 2018 [33]	3 points consecutive normal and breast cancer patients community & tertiary-centre study explicit exclusion of prior therapy	1 point age and menopausal status modelled subgroup by intrinsic subtype	2 points radiomic outcomes extracted blindly (software); no longitudinal outcome so limited follow-up;	6 points
Liu et al., 2021 [34]	4 points multicentre study tissue diagnosis confirmed controls = other subtypes within cohort	2 points multivariable model adjusts for tumour size, SUV and age	2 points outcome = IHC subtype (gold-standard) - blinded reading - no long-term follow-up	8 points
Araz et al., 2022 [35]	3 points consecutive patients single centre internal controls outcome absent at baseline	1 point only ER/PR tested vs. SUVs	2 points HR status from IHC - readers blinded - no follow-up	6 points
Jia et al., 2023 [36]	3 points clear inclusion and histology-proved internal controls outcome absent	1 point model includes MTV and clinicopathological factors but missing age or grade	2 points androgen receport status from pathology - blinded feature extraction - no follow-up	6 points
Hou et al., 2024 [37]	4 points prospectively maintained patient database PET protocol	1 point adjusted for molecular subtype no sociodemographics	2 points - pCR from surgery ≥24 mo DFS analysis but 6% lost	7 points
Hong et al., 2024 [38]	4 points pretreatment PET clear criteria consecutive cohort	1 point model includes MTV, subtype but is missing age or BMI	3 points pCR & PFS median follow-up 39 months censoring < 5%	8 points

Legend: NOS scores were calculated by the author based on the information reported in the included studies.

**Table A2:** Radiomics software reported in the included studies.

Name	Category	Modality Scope	Type/License	Notes	URL
PyRadiomics	Software (open-source/freeware)	Multimodality (CT/MR/PET/US)	Open-source (BSD-3-Clause)	Python library for radiomics feature extraction; supports DICOM SR TID 1500 output	https://github.com/AIM-Harvard/pyradiomics
LIFEx	Software (open-source/freeware)	PET, SPECT, MR, CT, US	Freeware (research use)	GUI application for radiomic feature calculation; published in Cancer Research (2018)	https://www.lifexsoft.org/
3D Slicer	Software (open-source/freeware)	Multimodality Via 3D Slicer	Open-source	Graphical interface to PyRadiomics inside 3D Slicer	https://github.com/AIM-Harvard/SlicerRadiomics
CGITA	Software (open-source/freeware)	Molecular imaging (PET/SPECT) also CT/MR	Open-source (MATLAB-based)	Texture analysis/radiomics for molecular images; original 2014 paper on PMC	https://pmc.ncbi.nlm.nih.gov/articles/PMC3976812/

This table lists only the software explicitly reported in the primary studies included in the review. All of them were accessed on 21 August 2025.

**Table A3:** Selected PET/CT radiomics studies in untreated BC.

Author	Objective	No. of Patients	Materials and Methods	Results	Conclusions
Ha et al. (2017) [32]	Evaluation of [18F]FDG PET/CT radiomics for LABC characterization and prediction of response to NAC	73	Retrospective study Image analysis: CGITA ver. 1.4 109 textural features Statistical analysis: MedCalc version 14.8.1 and R version 3. 2.3 Unsupervised Clustering Pearson Correlation; Hierarchical clustering (heatmaps); Kruskal–Wallis test; Chi-square test; Univariate and multivariate logistic regression; Kaplan–Meier curve and log-rank test; Univariate and multivariate Cox regression; Bonferroni correction;	TC II: had a pCR, increased SUVmax, and high intratumoral heterogeneity. TC I: Associated with an increased risk of recurrence. Significant radiomic features: SUVmax, MTV, TLG, and features specific to intratumoral heterogeneity (NL_EntropyGLCM, NL_HomogeneityGLCM, ZPGLSZM, Skewness) Notable differences between tumor clusters for radiomic parameters	Radiomic pretreatment correlates with Ki67, can predict pCR, risk of recurrence, and **response** to NAC.
Boughdad et al. (2018) [33]	The influence of age on metabolic parameters and radiomic features in breast tumors and healthy breast tissue. Biological variability in radiomic models	522	Retrospective study Image analysis: LIFEx software (www.lifexsoft.org) Metabolic markers: SUVmax, SUVmean, SUVpeak, MTV, TLG 4 histogram-based indices (HBI) SkewnessH, KurtosisH, EntropyH, EnergyH 31 TIs, of which 6 indices were considered robust: Homogeneity, Entropy, Gray-Level Run Length (LRE), Neighboring Gray-Level Dependence (SRE), Gray-Level Zone Length (LGZE), High Gray-Level Zone Length (HGZE) Statistical Analysis: IBM SPSS Statistics v22. ANOVA test; Bonferroni/Hochberg tests; Spearman correlation;	Age groups: PRE (under 45 years old), PERI (45 to under 55 years old), POST (55 to under 85 years old). Significant differences were found between metabolic markers (SUVmax, SUVmean, SUVpeak) and 12 TI (including homogeneity, SRE, HGZE), *p* < 0.05. Metabolic markers decrease with age. Homogeneity and LRE tend to increase. TI: Significant differences between groups for homogeneity, SRE, and HGZE (*p* < 0.05). Significant differences were observed in triple-negative tumors between PET and TI parameters (*p* < 0.05).	There are significant differences (SUV and TI) depending on age, especially in triple-negative carcinoma. Age should be considered as an additional variable in radiomics studies.
Liu et al. (2021) [34]	Using radiomics for the classification of breast cancer molecular subtypes	273	Retrospective study Image analysis: ImageJ 1.50i software (National Institute of Health, Bethesda, MD, USA) MATLAB, The MathWorks Inc., Natick, MA, USA Metabolic markers: SUVmax, SUVmean, SUVpeak, MTV, TLG 1710 radiomic features (855 PET-specific and 855 CT-based, respectively) Statistical analysis: R statistical software Wilcoxon rank-sum test; inter-feature correlation coefficient (R); Cox and LASSO regression; Rad-score; AUC; 10-fold cross-validation with 10 repetitions	Mean AUC: 0.856 (Luminal vs. Non-Luminal), 0.818 (HER2+ vs. HER2−), 0.888 (TN vs. Non-TN). Average accuracy: 0.864 (Luminal vs. Non-Luminal), 0.847 (HER+ vs. HER2−), 0.893 (TN vs. Non-TN). Average sensitivity: 0.801 (Luminal vs. Non-Luminal), 0.908 (HER2+ vs. HER2−), 0.933 (TN vs. Non-TN). Average Specificity: 0.905 (Luminal vs. Non-Luminal), 0.764 (HER+ vs. HER2−), 0.839 (TN vs. Non-TN)	Radiomics-based models can provide more information than classic PET/CT metabolic markers. Based on imaging phenotypes, radiomics can predict the molecular profile of breast carcinoma.
Araz et al. (2022) [35]	Establishing the status of hormone receptors in breast tumors using PET/CT radiomics	153	Retrospective study Image analysis: LIFEx (www.lifexsoft.org) Metabolic markers: SUVmin; SUVmax; SUVpeak and SUVmax, MTV and TLG respectively 42 radiomic features: first-order parameters-skewness; kurtosis; entropy-histo; energy, SHAPE-sphericity, compactness; second-order: GLCM homogeneity, GLCM energy, GLCM contrast, GLCM entropy, GLCM dissimilarity) higher-order: GLRLM (SRE, LRE, LGRE, HGRE, SRLGE, SRHGE, LRLGE, LRHGE, GLNU, RLNU, RP) GLZLM (SZE, LZE, LGZE, HGZE, SZLGE, SZHGE, LZLGE, LZHGE, GLNU, ZLNU, ZP) Coarseness, Contrast, Busyness Statistical analysis: WEKA 3.7 and SPSS 11.5 Mann–Whitney U test; binary logistic regression analysis; ROC; Hoeffding tree; J48; multilayer perceptron; 10-fold cross-validation; accuracy; F-measure; precision; recall; area under the precision-recall curve	By applying radiomic data analysis methods (binary regression and CorrelationAttributeEval), only 7 statistically relevant features remained: SUVmean, SUVmax, SUVpeak, GLZLM LZE, TLG, MTV, and GLRLM-GLNU. No statistically significant correlations were found between the radiomic features and hormone receptor status.	Radiomic features could not predict hormone receptor status. Patients who had hormone receptors had lower SUVs.
Jia et al. (2023) [36]	Prediction of androgen receptor expression based on clinico-pathological and PET/CT radiomic features	48	Retrospective study Image analysis: LIFEx v7.0.0 (www.lifexsoft.org) 80 radiomic features Metabolic markers: SUVmin, SUVmean, SUVmax, SUVstd, UVpeak. TLG, MTV Shape: Sphericity, Compacity, Volume-ml, Volume-vx Histogram: Skewness, Kurtosis, Entropy_log10, Entropy_log2, Energy GLCM: Homogeneity, Energy, Contrast, Correlation, Entropy_log10, Entropy_log2, Dissimilarity GLRLM: SRE, LRE, LGRE, HGRE, SRLGE, SRHGE, LRLGE, LRHGE, GLNU, RLNU, RP GLZLM: SZE, LZE, LGZE, HGZE, SZLGE, SZHGE, LZLGE, LZHGE, GLNU, ZLNU, ZP NGLDM: Coarseness, Contrast, Busyness Statistical analysis: IBM SPSS statistics version 26.0, Python version 3.11 (https://www.python.org), MedCalc software (MedCalc Software, Ostend, Belgium), and R version 4.2.1 (http://www.R-project.org) The radiomic features were integrated into a multivariate logistic regression model, which was subsequently evaluated using the ROC curve, the Hosmer-Lemeshow test, and DCA. Kolmogorov–Smirnov test, Levene’s test, *t*-test, Mann–Whitney U test, Chi-square test, Fisher’s test, Pearson correlation, 10-fold cross-validation	MTV is significantly associated with androgen receptor expression (*p* < 0.05) (an increased MTV value could be associated with negative receptor expression). Based on the logistic regression model, MTV, SHAPE-sphericityCT, and GLCM-contrastCT were included in the androgen receptor prediction model. The latter being a good predictor of androgen receptor status (OR = 9, *p* = 0.018). The model showed an AUC ROC of 0.832, sensitivity and specificity over 75%. The Hosmer-Lemeshow test indicated good calibration (*p* > 0.05).	The model built based on clinico-pathological data and PET/CT radiomic features can predict the presence of the androgen receptor in patients with breast carcinoma.
Hou et al. (2024) [37]	Evaluation of intratumoral and peritumoral radiomic features for assessing pCR response after NAC	190	Retrospective study Image analysis: 3D Slicer v4.11.20200930 and PyRadiomics (IBSI) After applying the Wavelet and Laplacian-Gaussian filters, 3864 radiomic features were obtained as follows: 1932 intratumoral and 1932 peritumoral. Statistical analysis: R software 4.3.1 and Python 3.7.9 Pearson correlation, Spearman correlation, *t*-test, LASSO, ORC-AUC and DeLong test, χ^2^, SMOTE. SVM, KNN, LR, NB Classifiers	10 radiomic features filtered through wavelet and Laplacian-Gaussian, then selected by LASSO: pet.intra.wavelet. HLH_firstorder_Skewness, ct.peri.wavelet. HHH_glrlm_LowGrayLevelRunEmphasis, ct.intra. wavelet. HLH_glcm_MCC, pet.intra.wavelet. HHL_firstorder_Skewness, pet.peri.wavelet. HHH_firstorder_10Percentile, ct.intra.wavelet. LHL_glcm_Idn, pet.peri.wavelet. HHL_glrlm_LowGrayLevelRunEmphasis, ct.peri.wavelet. LLH_firstorder_RootMeanSquared, pet.peri.wavelet. LHH_glszm_ZoneEntropy, pet.intra.wavelet. The features showed robustness to segmentation (ICC > 0.75) and were validated with SMOTE. Intratumoral and peritumoral SVM had the best prediction of pCR, with an AUC of 0.83, significantly outperforming KNN, LR, and NB. The molecular subtype models maintained high performance (AUC 0.86–0.92).	Combining intra- and peritumoral features improves performance in pCR prediction.
Hong et al. (2024) [38]	The main objective was to demonstrate that NHOC and NHOP can predict progression-free survival and rPC in NAC for patients with breast carcinoma.	135	Retrospective study Image analysis: LIFEx v. 7.6.0 (www.lifexsoft.org) according to (IBSI) Classic metabolic markers SUVmax/peak, MTV, TLG Distance-derived radiomic markers: NHOCmax/peak and NHOPmax/peak Statistical Analysis: MedCalc Statistical v. 22.021 Mann–Whitney U, Kruskal–Wallis, Spearman, ROC-AUC, logistic regression, Cox, Kaplan–Meier, log-rank test	NHOCmax AUC = 0.71 (95% CI: 0.59–0.80); NHOCpeak AUC = 0.69 significantly better than SUVmax, SUVpeak, MTV, TLG. With low NHOCmax and NHOCpeak values (“hot spot” near the center), the chance of pCR is higher. MTV and NHOCpeak demonstrated independence from the rest of the markers. NHOCpeak mic (≤0.27)—92% progression-free survival at 5 years compared to 67%.	NHOCpeak can predict pCR and response to NAC. Increased NHOCpeak is associated with a poorer prognosis.

Legend: LABC—locally advanced breast cancer; NAC—neoadjuvant chemotherapy; CGITA—Chang-Gung Memorial Hospital, Taiwan; TC—tumor cluster; pCR—pathological complete response; SUVmax (maximum standardized uptake value); SUVmean (mean value of the standardized uptake value); SUVpeak (average standardized uptake value within a 1 mL sphere at a position that maximizes the average value within the sphere); MTV (metabolic tumor volume), TLG (SUVmean x MTV); NL_EntropyGLCM (Non-Localized Entropy—Gray-Level Co-occurrence Matrix); NL_HomogeneityGLCM (Non-Localized Homogeneity—Gray-Level Co-occurrence Matrix); ZPGLSZM (Zone Percentage—Gray-Level Size Zone Matrix); AUC- area under the ROC curve; TI- textural index; GLRLM [SRE(Short run emphsis); LRE (Long run emphasis); LGRE (Low gray level run emphasis); HGRE (High gray level run emphasis); SRLGE (short run low gray level emphasis); SRHGE (short run high gray level emphasis); LRLGE (Long run low gray level emphasis); LRHGE (Long run high gray level emphasis); GLNU (gray level non uniformity for run); RLNU (run length non uniformity); RP (run percentage)]; GLZLM—[SZE (short zone emphasis); LZE (long zone emphasis); LGZE (low gray level zone emphasis); HGZE (high gray level zone emphasis); SZLGE (short zone low gray level SZHGE (short zone high gray level emphasis); LZLGE (long zone low gray level emphasis); LZHGE (long zone high gray level emphasis); GLNU (gray level non uniformity for zone); ZLNU (zone length non uniformity); ZP (zone percentage)]; ROC curve—receiver operating characteristic curve; DCA—decision curve analysis; GLCM_ContrastCT—contrast on CT from the gray-level co-occurrence matrix; SVM—support vector machine; KNN—k-nearest neighbors; LR—logistic regression; SMOTE—synthetic minority oversampling technique; NHOCmax—the normalized distance between the voxel with SUVmax and the geometric center of the tumor; NHOCpeak—the normalized distance between the center of the SUVpeak volume and the geometric center of the tumor; NHOPmax—the normalized distance between the voxel with SUVmax and the tumor perimeter; NHOPpeak—the normalized distance between the center of the SUVpeak volume and the tumor perimeter.
